# Design and Analysis of Multi-User Faster-Than-Nyquist-DCSK Communication Systems over Multi-Path Fading Channels

**DOI:** 10.3390/s22207837

**Published:** 2022-10-15

**Authors:** Mohamed Dawa, Marijan Herceg, Georges Kaddoum

**Affiliations:** 1 ETS, LaCIME Laboratory, University of Québec, 1100 Notre-Dame West, Montreal, QC H3C 1K3, Canada; 2 Department of Communications, Faculty of Electrical Engineering, Computer Science and Information Technology, 31000 Osijek, Croatia

**Keywords:** chaos-based communication systems, differential chaos shift keying, Faster-Than-Nyquist, interference, multi-user, sampling rate

## Abstract

In this paper, we present a new multi-user chaos-based communication system using Faster-than-Nyquist sampling to achieve higher data rates and lower energy consumption. The newly designed system, designated Multi-user Faster Than Nyquist Differential Chaos Shift Keying (MU-FTN-DCSK), uses the traditional structure of Differential Chaos Shift Keying (DCSK) communication systems in combination with a filtering system that goes below the Nyquist limit for data sampling. The system is designed to simultaneously enable transmissions from multiple users through multiple sampling rates resulting in semi-orthogonal transmissions. The design, performance analysis, and experimental results of the MU-FTN-DCSK system are presented to demonstrate the utility of the newly proposed system in enabling multi-user communications and enhancing the spectral efficiency of the basic DCSK design without the addition of new blocks. The MU-FTN-DCSK system presented in this paper demonstrates spectral gains for one user of up to 23% and a combined gain of 25% for four (U=4) users. In this paper, we present a proof of concept demonstrating a new degree of freedom in the design of Chaos-based communication systems and their improvement in providing wireless transmissions without complicated signal processing tools or advanced hardware designs.

## 1. Introduction

Higher data rates and energy efficiency are the core goals when designing new chaos-based communication systems. These goals originate from different aspects, like introducing the new communication standards (4G&5G) and the new challenges related to spectrum distribution to enable massive numbers of devices to access the network. Chaos-based systems, used as spread spectrum communication systems, were proven to be a great candidate for communications of IoT devices that constitute a considerable portion of the newly introduced standards [1].

In this vein, among the significant motives for improving chaos-based systems is the inherent limitations of non-coherent chaos-based systems and the rapid development of new methods that can profit the design of new highly performing chaos-based schemes. In the literature, the non-coherent scheme called Differential Chaos Shift Keying (DCSK) [2] has received significant attention in developing new chaos-based schemes, given its simple detection structure and its proven ability to mitigate the effect of fading channels [3]. It is also one of the most adaptive schemes and has been extensively studied over the last two decades and has seen numerous upgrades in its basic symbol structure. In this sense, various signal processing approaches have been developed to improve its data rate, spectral efficiency, transmit energy efficiency, and Bit-Error-Rate (BER). Among the most notable emerging modulation schemes, index modulation has been primarily explored, given its potential to encode data more optimally and therefore ameliorate the performances of DCSK systems. Index modulation (IM) was first studied for Orthogonal Frequency Division Multiplexing (OFDM) in [4], where it was shown that IM could be used for more than just the transmit antennas of MIMO systems. IM was then applied to DCSK and Short Reference DCSK (SR-DCSK) using walsh codes in [5] which showed that the application of Code-Index Modulation (CIM) results in a considerable performance enhancement. Walsh codes are combined with a natural number-based mapping method in [6] to improve the data rate of the simple Code-Index Modulation DCSK (CIM-DCSK) while using the same resources. A combination of multiple modulation techniques, such as OFDM, vector modulation, and index modulation, has been proposed as a method for improving the data rate and spectral efficiency of chaos-based systems. The data rate was addressed in [7] with the introduction of superposition coding to Pulse-Position-Modulation DCSK (PPM-DCSK) to enhance multi-user download rates. Permutation index differential chaos shift keying (PI-DCSK) and differential permutation index differential chaos shift keying (DPI-DCSK) modulations were presented in [8,9], where a permutation is used to encode additional data with a list of codes referring to a series of chaotic references. Sparse codes were used similarly to achieve higher spectral efficiency for the Multi-carrier DCSK systems in [10]. In [11], time-reversal transform is combined with the DCSK design to improve the BER and spectral performances given a land mobile satellite channel model. According to [12], optimal finger selection based on a genetic algorithm can be used to enhance the performance of the MMSE receiver. Orthogonal chaotic generators (OCG) were combined with Walsh codes in [13] to enable multi-user transmissions and achieve higher spectral efficiency than the DCSK systems. General Carrier Indexing (GCI), which uses carrier indexing in a more general and arbitrary form to reduce the number of indexes required for carriers and data bits, was used in [14] to provide higher spectral efficiency than conventional index-based DCSK systems. Combining M-ary modulation with index modulation and multi-carrier transforms in [15] increased data rate but at the expense of much higher complexity. Amplitude Phase Shift Keying (APSK) was applied instead of Quadrature Amplitude modulation (QAM) to the DCSK scheme in [16] in order to enhance the Peak-to-Average Power Ratio (PAPR) and the robustness against channel estimation errors. The authors in [17] used M-ary phase shift keying (MPSK) in combination with parallelly concatenated index modulation. They integrated it with carrier interferometry (CI) codes into the OFDM-DCSK to reach a satisfactory quality of service (QoS) and low PAPR for all users. Precoding was used in [18] to encode the data of Multi-Carrier M-ary Chaotic Vector Cyclic Shift Keying (MC-M-CVCSK) to combat the noise and interferences caused by the channel.

Meanwhile, one of the emerging and most efficient signal processing techniques for improving communication systems’ throughput is Faster than Nyquist (FTN), which defies the limit set by Nyquist for inter-symbol spacing, thus resulting in data rate improvements. The approach has been extensively studied for possible time and frequency domains superpositions [19]. In [20], the FTN approach is proven to provide an increased data rate of 23%, when the symbol spacing is at a specific limit, named the Mazo limit, where the reduction in the spacing between symbols does not affect the BER performance. The reduction can be applied in time and frequency, providing two degrees of freedom for enhancing the performance of communication systems. FTN signaling has been studied extensively, especially for optimal detection of signal-spacings lower than the Mazo-limit [21], while traditional detection algorithms, like the Maximum Likelihood Sequence Estimation (MLSE) and Viterbi algorithm, provide decent performance for a limited decrease in sampling rates, their detection optimality is limited to a short increase of symbol rate. In addition, they induce an additional computational complexity for high-order constellations. New algorithms have been proposed to tackle the computation time issue. For instance, in [22], to achieve a sub-optimal detection while maintaining a polynomial solving time, the sequence estimation is based on the semidefinite relaxation (SDR) technique to achieve a sub-optimal detection. In [23], output-retainable convolutional codes (ORCCs) were used for channel memorization to reduce the decoding complexity. A new approach based on a deep learning-assisted sum-product detection algorithm (DL-SPA) was presented in [24] for faster detection convergence.

Moreover, lately, the key research goals on FTN-based modulations focus on reaching higher orders of magnitude in the frequency domain, designing optimal filters in the time domain, Inter-Symbol-interference (ISI) and Inter-Carrier-Interference (ICI) reduction, and the elaboration of a general FTN theory. A comparative analysis of Frequency-domain equalization (FDE) and time-domain equalization (TDE) was carried out in [25] where it was shown that TDE outperforms FDE, especially for ISI elimination; however, this comes at the cost of increased complexity. In [26], FDE was improved using Expectation Propagation (EP) to achieve a performance similar to that of the Bahl Cocke Jelinek and Raviv (BCJR) receiver [27] with less complexity. However, all related FTN works introduce additional algorithmic or hardware-related computation requirements. To date, no simple yet efficient modulation scheme has been introduced in the literature. More complexity is expected to be introduced going forward with the research on a generalized FTN theory and its optimal detection. The main goal of this paper is to introduce a design that combines the architectural simplicity of DCSK and the benefits of a transform that enables a spectral gain namely, FTN. Accordingly, the spectrum would be used less, depending on the level of the filtering rate, ranging from a maximum value at Nyquist rate (ρ=1) to zero (ρ=0), for which no spacing would be introduced between symbols. This would enable spectral gains ranging from zero to 100% depending on the BER requirements.

This paper introduces a novel multi-user FTN-based DCSK system where a combination of FTN filtering and non-coherent detection allows better usage of the available spectrum for the DCSK systems. Moreover, the new system enables multi-user access using the same mechanism that enables spectral efficiency gain, i.e., the FTN sampling. The MU-FTN-DCSK enables multi-user access via different sampling rates with a gain in spectral efficiency for all the users simultaneously transmitting within the FTN regime. The BER results prove the system’s capabilities for various sampling rates and spreading factors. The contributions of this paper are listed as follows:

1. Designing the Multi-user Faster-Than-Nyquist system that enables access for users with different sampling rates, all transmitting in an FTN regime.
2. Derivation of the theoretical BER expression for the proposed system over Multi-path Rayleigh Fading channels.
3. Performance analysis of the designed system, via monte-Carlo simulation, over the AWGN and Rayleigh fading channels for different system parameters and configurations.

The rest paper is structured as follows. In Section 2, the MU-FTN-DCSK system is presented. Section 3 derives the BER expressions of the system. In Section 4, the simulation results of the MU-FTN-DCSK system are presented along with their analysis. The paper is concluded in Section 5, where we highlight the most important findings related to the newly introduced system.

## 2. System Design

The FTN-DCSK system combines a sinc filter functioning at a sub-Nyquist sampling rate with the basic transmit-receive DCSK design. The system is described in this section, where the schematic and mathematical representations of the transmitted signals are provided. The FTN prospect reflects on the possibility of enabling filtering on sub-Nyquist rates, which reduces the separation between successive transmissions to less than the minimum distance guaranteeing orthogonality. The belief proceeding FTN was that such reduction would be disastrous on signal transmission following the theorems of Shannon and Nyquist for optimal filter design and sampling. It is only through the work of T.Mazo [20] that this belief was questioned, where in their work, he proved that a 401 Hz/bit/s was sufficient for a sub-Nyquist filter to guarantee the same Euclidean distance offered by an ideal Sinc filter in regular orthogonal transmissions. As a result, with FTN, compared to a regular orthogonal pulse with a separation period *T*, the Mazo limit is ρ = 0.802 *T*.

Moreover, the DCSK system is based on transmitting a chaotic sequence (reference) of length β and its product with the data signal in two successive time slots. The detection is based on differential detection, where the incoming signal is delayed by β. Then the product of the two sequences in the different time slots is taken, followed by a summation over the β duration. Finally, the sign is taken to determine the transmitted data symbol.

### 2.1. FTN-DCSK

**Figure 1:**
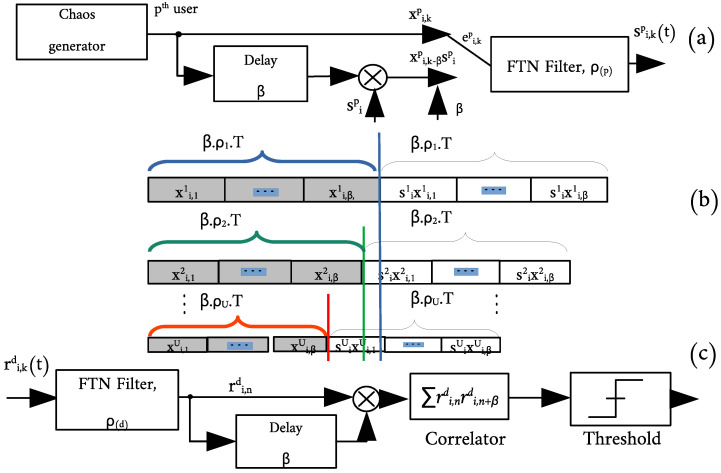
Block diagram of the MU-FTN-DCSK communication system, (**a**) Random *p*th user’s transmitter, (**b**) Frame shapes for different users with different sampling parameters, (**c**) Receiver structure for the desired user.

The FTN-DCSK system is constructed using a Sinc filter with a sub-Nyquist sampling rate to encode the chaotic sequence at the output of the DCSK system, as shown in the block diagram in Figure 1a. We use the normalized Chebychev map for chaos generation

(1) $$e_{i,k}^{p}=\left\{\begin{array}{cc} x_{i,k}^{p} & \mathrm{for}\;2i\beta +1<k\leq (2i\beta +1)\beta \\ b_{i}^{p}x_{i,k-\beta }^{p} & \mathrm{for}\;(2i\beta +1)\beta <k\leq (i+1)2\beta , \end{array}\right.$$

where $x_{i,k}^{p}$ is the *i*th state for *i*th symbol and *k* is the index of chaotic sample, for the *p*th user, with a spreading factor β for the reference and data bearing signals, and the $b_{i}^{p}\in \left[-1,1\right]$ are the data bits. We use a normalized Sinc filter $g_{\rho_{\left(p\right)}}^{k}$ to encode the chaotic sequences with a different sampling parameter $\rho_{\left(p\right)}$ for every user, expressed as

(2) $$\begin{array}{c} g_{\rho_{\left(p\right)}}^{k}\left(t\right)=\sqrt{\rho_{\left(p\right)}}sinc\left(t-k.\rho_{\left(p\right)}.T\right), \end{array}$$

where *k* is the chaotic sample being encoded, $\rho_{p}$ is the sampling parameter for *p*th user, and *T* is the typical Nyquist period. The multiplication by the sampling parameter’s square root is for normalizing the filters at both ends of the transmission. As a result, the continuous-time transmitted signal $s_{i,k}^{p}$(t), for the *p*th user, has the following form

(3) $$\begin{array}{c} s_{i,k}^{p}\left(t\right)=e_{i,k}^{p}g_{\rho_{\left(p\right)}}^{k}\left(t\right). \end{array}$$

For detection, the Sinc filter is first applied at the receiver, followed by the DCSK detection scheme. The multi-user system design is shown in Figure 1, where a different FTN sampling parameter is used for each user. The detection is based on selecting the desired user’s signal by passing the combined signal, summed in the channel, through the filter with the designated sampling parameter $\rho_{d}$, followed by the delay loop and the product and summation blocks.

The index *d* is used to separate the symbol of the desired user from the rest of the users transmitting in the same channel. Hence, the received signal $r_{i,k}^{d}\left(t\right)$ during 2β samples, for the desired user with a sampling parameter $\rho_{\left(d\right)}$, corresponding to the *k*th sinc pulse of the *i*th transmitted symbol, is expressed as

(4) $$\begin{array}{cc} r_{i,k}^{d}\left(t\right)= & \sum_{l=1}^{L}\alpha_{i,l}^{d}s_{i,k-\tau_{i,l}}^{d}\left(t\right)+\sum_{k^{\prime }=-\infty }^{\infty }\sum_{p=1}^{U-1}\sum_{l^{\prime }=1}^{L}\alpha_{i^{\prime },l^{\prime }}^{p}s_{i^{\prime },k^{\prime }-\tau_{i^{\prime },l^{\prime }}}^{p}\left(t\right)+W\left(t\right),\;i^{\prime }=\left\lfloor \frac{k^{\prime }}{2\beta }\right\rfloor , \end{array}$$

where *U* is the total number of users, $\alpha_{i^{\prime },l^{\prime }}^{p}$ is the *p*th user’s channel coefficient over the $l^{\prime }$ th channel tap, and l={1,…,L}, $\tau_{i^{\prime },l^{\prime }}$ is the $\left(l^{\prime }\right)$ th channel tap delay for the $\left(i^{\prime }\right)$ th symbol. Similarly, $\alpha_{i,l}^{d}$ is the desired user’s channel coefficient over the *l*th channel tap, l={1,…,L}, $\tau_{i,l}$ is the *l*th channel tap delay for the *i*th symbol, ⌊.⌋ is the floor operator, and *W* is the noise signal.

The detection is based on applying the matched filter to the received signal $r_{i,k}^{d}\left(t\right)$ in order to retrieve the different $e_{i,k}^{d}$ samples and is expressed as

(5) $$\begin{array}{c} r_{i,n}^{d}=\langle r_{i,k}^{d}\left(t\right),g_{\rho_{\left(d\right)}}^{n}\left(t\right)\rangle . \end{array}$$

〈,〉 is the dot product.

For filters with different sampling factors $\rho_{\left(d\right)}$ at reception for the desired user and $\rho_{\left(p\right)}$ for any random user with a different sampling parameter, the detection matrix is derived as follows,

(6) $$\begin{array}{cc} G_{k,n}^{p}= & \langle g_{\rho_{\left(p\right)}}^{k}\left(t\right),g_{\rho_{\left(d\right)}}^{n}\left(t\right)\rangle \\ = & \int_{-\infty }^{\infty }g_{\rho_{\left(p\right)}}^{k}\left(t\right).\bar{g_{\rho_{\left(d\right)}}^{n}\left(t\right)}, \end{array}$$

by applying The Plancherel transform for integral substitution, we obtain

(7) $$\begin{array}{cc} G_{k,n}^{p}= & \int_{-\infty }^{\infty }e^{-j\omega k\rho_{\left(p\right)}.T}G_{T}\left(\omega \right).\bar{e^{-j\omega n\rho_{\left(d\right)}.T}G_{T}\left(\omega \right)}d\omega \\ = & \int_{-\infty }^{\infty }e^{-j\omega (k\rho_{\left(p\right)}-n\rho_{\left(d\right)}).T}{\left|G_{T}\left(\omega \right)\right|}^{2}d\omega , \end{array}$$

where $G_{T}\left(\omega \right)$ is the Fourier transform of the regular sinc filter. The Fourier transform is applied again with a variable change θ=ωT, which gives

(8) $$\begin{array}{cc} G_{k,n}^{p}= & \frac{1}{2\pi }\int_{-\pi }^{\pi }e^{-j\theta (k\rho_{\left(p\right)}-n\rho_{\left(d\right)})}d\theta \\ = & \sqrt{\rho_{\left(p\right)}\rho_{\left(d\right)}}sinc\left(k\rho_{\left(p\right)}-n\rho_{\left(d\right)}\right). \end{array}$$

For the same sampling parameter $\rho_{\left(d\right)}$ in transmission and reception, the dot product of the two vectors obtained at the output of the matched filters, as shown in [28], is expressed as,

(9) $$\begin{array}{c} \langle g_{\rho_{\left(d\right)}}^{k}\left(t\right),g_{\rho_{\left(d\right)}}^{n}\left(t\right)\rangle =\rho_{\left(d\right)}sinc\left(\rho_{\left(d\right)}\left(k-n\right)\right). \end{array}$$

We consider a dissimilar multi-path Rayleigh channel model with *L* paths and a PDF expressed as

(10) $$\begin{array}{c} f\left(\alpha_{i,l}^{p}\right)=\frac{\alpha_{i,l}^{p}e^{-{\alpha_{i,l}^{p}}^{2}/\sigma^{2}}}{\sigma^{2}},\;\;\alpha_{i,l}^{p}>0, \end{array}$$

where $\alpha_{i,l}^{p}$ is the channel gain and σ is its standard deviation. The overall energy over a symbol duration is

$$\sum_{l=1}^{L}E\left(\alpha_{i,l}^{p}\right)=1,$$

here $E(\alpha_{i,l}^{p})$ is the energy level for the *l*th path for both cases of similar and dissimilar power distribution over the channel paths.

The following assumptions are considered for transmission: (1) the coefficients of the channel $\alpha_{i,l}^{p}$ are independent and identically distributed (iid); (2) the bit energy is constant over the symbol duration of length 2β; (3) the delay spread of the channel $D_{s}$ is neglected for the same reason, ensuring that the channel introduces no ISI, i.e., $D_{s}<<\beta$.

### 2.2. Spectral Efficiency

For a single user, the length of an MU-FTN-DCSK symbol is given as (2$\beta \rho_{\left(d\right)}T$). Hence, we obtain the percentage of spectral gain $S_{e}$ expressed as,

(11) $$S_{e}=\left(\frac{1}{\rho_{\left(d\right)}2\beta T}-\frac{1}{2\beta T}\right)\times 100\%.$$

The spectral gain is then simplified to the following expression

(12) $$S_{e}=\left(\frac{1-\rho_{\left(d\right)}}{\rho_{\left(d\right)}}\right)\times 100\%.$$

For the total spectral gain for *U* users, we sum over the total number of users as follows

(13) $$S_{e}=\sum_{p=1}^{U}\left(\frac{1-\rho_{\left(p\right)}}{\rho_{\left(p\right)}}\right)\times 100\%.$$

Given this expression, we know that the system’s spectral efficiency depends on the total number of users and their individual sampling parameters. Therefore, to reach a maximal spectral efficiency, one would have to increase the number of users to maximum while trying to minimize their sampling parameters concerning the desired BER performance.

## 3. Performance Analysis

This section derives the BER expression for the proposed MU-FTN-DCSK system over multi-path Rayleigh fading channels. Following the design presented in Figure 1, the Probability Density Function (PDF) of the received signal follows an exponential distribution expressed as

(14) $$f\left(\gamma_{i,l}^{p}\right)=\sum_{l=1}^{L}\frac{\tau_{i,l}^{p}}{{\bar{\gamma }}_{i,l}^{p}}e^{-\frac{\gamma_{i,l}^{p}}{{\bar{\gamma }}_{i,l}^{p}}},$$

where $\gamma_{i,l}^{p}$ is the channel gain, ${\bar{\gamma }}_{i,l}^{p}$ is its averaged value, and $\tau_{i,l}^{p}$ being the weight for the *i*th symbol duration over the *l*th path and is expressed as

(15) $$\tau_{i,l}^{p}=\prod_{j=1,\;j\neq l}^{l}\frac{\gamma_{l}}{\gamma_{l}-\bar{\gamma_{j}}}.$$

Hence, considering [28] (Eq. 3.3), the result in (8), and the normalized filters for different users, the received signal $r_{i,n}^{d}$ post filtering during the first β samples, i.e., the *n*th sample of the reference signal, for the desired user with a sampling parameter $\rho_{\left(d\right)}$ is expressed as:

(16) $$\begin{array}{cc} r_{i,n}^{d}= & \sum_{k=-2}^{\beta +2}\sum_{l=1}^{L}\alpha_{i,l}^{d}x_{i,k-\tau_{i,l}}^{d}\rho_{\left(d\right)}sinc\left(\rho_{\left(d\right)}\left(k-n\right)\right)+\sum_{k^{\prime }=-\infty }^{\infty }\sum_{p=1}^{U-1}\sum_{l^{\prime }=1}^{L}\alpha_{i^{\prime },l^{\prime }}^{p}x_{i^{\prime },k^{\prime }-\tau_{i^{\prime },l^{\prime }}}^{p}\\ \; & \times \sqrt{\rho_{\left(p\right)}\rho_{\left(d\right)}}sinc\left(k^{\prime }\rho_{\left(p\right)}-n\rho_{\left(d\right)}\right)+W_{n}. \end{array}$$

$W_{n}$ is the noise signal received during the same *i*th symbol duration. It is assumed that only two samples prior to and after the desired signal can influence it, and thus the value of k={−2,β+2}. Similarly, the value of $k^{\prime }\in \left\{-\infty ,\infty \right\}$ is used to express the 2β samples transmitted by any other random user asynchronously. The operator ⌊.⌋ in (4) expresses the sampling of 2β sample at the moment of detection.

Similarly, the data-bearing signal, received during the second β samples is expressed by multiplying the signal of the desired user form (16) by the data bits $s_{i}^{d}$.

Due to the asynchronous nature of the system, both the reference and data-bearing signals only assume the chaos in the expression of other users’ signals so that generality is not lost… The assumption does not impact the derivation of the BER expression from a statistical perspective since $E|e_{i,k}^{p}|=1$. Hence, the decision variable for the desired user’s *i*th transmitted symbol for the MU-FTN-DCSK system is derived as,

(17) $$\begin{array}{cc} D_{i}= & \sum_{n=1}^{\beta }r_{i,n}^{d}*r_{i,n+\beta }^{d}\\ = & \sum_{n=1}^{\beta }(\sum_{k=-2}^{\beta +2}\sum_{l=1}^{L}\alpha_{i,l}^{d}x_{i,k-\tau_{i,l}}^{d}\rho_{\left(d\right)}sinc\left(\rho_{\left(d\right)}\left(k-n\right)\right)+\sum_{p=1}^{U-1}\sum_{l_{1}^{\prime }=1}^{L}\sum_{k^{\prime }=-\infty }^{\infty }\alpha_{i^{\prime },l_{1}^{\prime }}^{p}x_{i^{\prime },k^{\prime }-\tau_{i^{\prime },l_{1}^{\prime }}}^{p}\sqrt{\rho_{\left(p\right)}\rho_{\left(d\right)}}\\ & \times sinc\left(k\rho_{\left(p\right)}-n\rho_{\left(d\right)}\right)+W_{n})\times (\sum_{k=-2}^{\beta +2}\sum_{l^{\prime }=1}^{L}\alpha_{i,l^{\prime }}^{d}s_{i}^{d}x_{i,k-\tau_{i,l^{\prime }}}^{d}\rho_{\left(d\right)}sinc\left(\rho_{\left(d\right)}\left(k-n\right)\right)\\ & +\sum_{p^{\prime }=1}^{U-1}\sum_{l_{2}^{\prime }=1}^{L}\sum_{k^{\prime }=-\infty }^{\infty }\alpha_{i^{\prime },l_{2}^{\prime }}^{p^{\prime }}x_{i^{\prime },k^{\prime }-\tau_{i^{\prime },l_{2}^{\prime }}}^{p^{\prime }}\times \sqrt{\rho_{\left(p^{\prime }\right)}\rho_{\left(d\right)}}sinc\left(k\rho_{\left(p^{\prime }\right)}-n\rho_{\left(d\right)}\right)+W_{n+\beta }), \end{array}$$

The desired signal is then the product of the chaotic terms having the index of the desired user *d* and the filter with the desired user’s sampling parameter $\rho_{\left(d\right)}$, resulting in the following signal

(18) $$\begin{array}{cc} U_{i}= & \sum_{n=1}^{\beta }\sum_{k=-2}^{\beta +2}\sum_{l=1}^{L}s_{i}^{d}{\left(\alpha_{i,l}^{d}\right)}^{2}{\left(x_{i,k-\tau_{i,l}}^{d}\right)}^{2}\rho_{\left(d\right)}^{2}\times sinc^{2}\left(\rho_{\left(d\right)}\left(k-n\right)\right), \end{array}$$

The cross-product of the chaotic terms in (17) results in interferences expressed as

(19) $$\begin{array}{c} \left.\begin{array}{ccc} I_{i} & = & \sum_{n=1}^{\beta }\sum_{k=-2}^{\beta +2}\sum_{k^{\prime }=-\infty ,k^{\prime }\neq k}^{\infty }(\sum_{l=1}^{L}\sum_{l^{\prime }=1}^{L}s_{i}^{d}\alpha_{i,l}^{d}\alpha_{i,l^{\prime }}^{d}x_{i,k-\tau_{i,l}}^{d}x_{i,k^{\prime }-\tau_{i,l^{\prime }}}^{d}\rho_{\left(d\right)}^{2}sinc\left(\rho_{\left(d\right)}\left(k-n\right)\right)\\ \; & \; & \times sinc(\rho_{\left(d\right)}\left(k^{\prime }-n\right)) \end{array}\right\}\mathrm{ISI}\\ \begin{array}{ccc} \; & + & \sum_{l=1}^{L}\sum_{l^{\prime }=1}^{L}\sum_{p=1}^{U-1}\alpha_{i,l}^{d}\left(1+s_{i}^{d}\right)\alpha_{i^{\prime },l_{1}^{\prime }}^{p}\sqrt{\rho_{\left(p\right)}}\times \rho_{\left(d\right)}^{3/2}x_{i,k-\tau_{i,l}}^{d}x_{i^{\prime },k^{\prime }-\tau_{i^{\prime },l^{\prime }}}^{p}sinc\left(\rho_{\left(d\right)}\left(k-n\right)\right)\\ \; & \times & sinc\left(k^{\prime }\rho_{\left(p\right)}-n\rho_{\left(d\right)}\right)+\sum_{p=1}^{U-1}\sum_{p^{\prime }=1}^{U-1}\sum_{l_{1}^{\prime }=1}^{L}\sum_{l_{2}^{\prime }=1}^{L}\alpha_{i_{1}^{\prime },l_{1}^{\prime }}^{p}\alpha_{i_{2}^{\prime },l_{2}^{\prime }}^{p^{\prime }}\sqrt{\rho_{\left(p\right)}\rho_{\left(p^{\prime }\right)}}\rho_{\left(d\right)}\\ \; & \times & x_{i_{1}^{\prime },k^{\prime }-\tau_{i_{1}^{\prime },l_{1}^{\prime }}}^{p}x_{i_{2}^{\prime },k^{\prime }-\tau_{i_{2}^{\prime },l_{2}^{\prime }}}^{p^{\prime }}sinc\left(k\rho_{\left(p\right)}-n\rho_{\left(d\right)}\right)\times sinc\left(k\rho_{\left(p^{\prime }\right)}-n\rho_{\left(d\right)}\right)) \end{array}\}\mathrm{IUI} \end{array}$$

The first term in (19) is the ISI, which occurs because of the reduced distances between samples due to the nature of the FTN filter. The remaining part is the inter-user interference (IUI) for different chaos generators and sampling parameters. The noise term is expressed as

(20) $$\begin{array}{cc} N_{i}= & \sum_{n=1}^{\beta }(W_{n-\beta }(\sum_{k=-2}^{\beta +2}\sum_{l=1}^{L}\alpha_{i,l}^{d}x_{i,k-\tau_{i,l}}^{d}\rho_{\left(d\right)}\times sinc\left(\rho_{\left(d\right)}\left(k-n\right)\right)+\sum_{p=1}^{U-1}\sum_{k^{\prime }=-\infty }^{\infty }\sum_{l_{1}^{\prime }=1}^{L}\alpha_{i^{\prime },l_{1}^{\prime }}^{p}s_{i^{\prime }}^{p}\\ & \times \sqrt{\rho_{\left(p\right)}\rho_{\left(d\right)}}x_{i^{\prime },k^{\prime }-\tau_{i^{\prime },l_{1}^{\prime }}}^{p}sinc\left(k\rho_{\left(p\right)}-n\rho_{\left(d\right)}\right))\\ & +W_{n}(\sum_{k=-2}^{\beta +2}\sum_{l=1}^{L}\alpha_{i,l}^{d}s_{i}^{d}x_{i,k-\tau_{i,l}}^{d}\rho_{\left(d\right)}sinc\left(\rho_{\left(d\right)}\left(k-n\right)\right)+\sum_{p^{\prime }=1}^{U-1}\sum_{k^{\prime }=-\infty }^{\infty }\sum_{l_{2}^{\prime }=1}^{L}\alpha_{i^{\prime },l_{2}^{\prime }}^{p^{\prime }}s_{i^{\prime }}^{p^{\prime }}x_{i^{\prime },k^{\prime }-\tau_{i^{\prime },l_{2}^{\prime }}}^{p^{\prime }}\\ & \times \sqrt{\rho_{\left(p^{\prime }\right)}\rho_{\left(d\right)}}sinc\left(k\rho_{\left(p^{\prime }\right)}-n\rho_{\left(d\right)}\right))+W_{n}W_{n-\beta }). \end{array}$$

In this paper, it is assumed that the sum of β chaotic samples is equal to,

(21) $$E_{b}=2\sum_{k=1}^{\beta }x_{i,k}^{2}$$

Hence, the mean value of the desired signal is given as

(22) $$\begin{array}{cc} E= & \frac{1}{2}\rho_{\left(d\right)}^{2}\frac{E_{b}}{\beta }\sum_{n=}^{\beta }\sum_{k=-2}^{\beta +2}\sum_{l=1}^{L}{\left(\alpha_{i,l}^{d}\right)}^{2}sinc^{2}\left(\rho_{\left(d\right)}\left(k-n\right)\right). \end{array}$$

Since the chaotic sequences are generated with zero mean ($E(x_{i,k})=0$) and using (21), the variance of the individual chaotic chips is given as,

(23) $$var\left(x_{i,k}^{2}\right)=\frac{E_{b}^{2}}{4\beta }$$

Hence, the variances of the interference terms in (19) can be split as the ISI, with variance $V_{I_{1}}$, the interference to the desired user, with variance $V_{I_{2}}$, and the interference among all other users, with variance $V_{I_{3}}$ as

(24) $$\begin{array}{cc} V_{I_{i_{1}}}= & \frac{E_{b}^{2}}{4\beta^{2}}\sum_{n=1}^{\beta }\sum_{k=-2}^{\beta +2}\sum_{k^{\prime }=-\infty }^{\infty }\sum_{l=1}^{L}\sum_{l^{\prime }=1}^{L}{\left(\alpha_{i,l}^{d}\alpha_{i,l^{\prime }}^{d}\right)}^{2}\\ \; & \times \rho_{\left(d\right)}^{4}sinc^{2}\left(\rho_{\left(d\right)}\left(k-n\right)\right)sinc^{2}\left(\rho_{\left(d\right)}\left(k^{\prime }-n\right)\right)\\ V_{I_{i_{2}}}= & \frac{E_{b}^{2}}{2\beta^{2}}\sum_{p=1,p\neq d}^{U}\sum_{n=1}^{\beta }\sum_{k=-2}^{\beta +2}\sum_{k^{\prime }=-\infty }^{\infty }\sum_{l=1}^{L}\sum_{l^{\prime }=1}^{L}{\left(\alpha_{i,l}^{d}\alpha_{i^{\prime },l^{\prime }}^{p}\right)}^{2}\rho_{\left(p\right)}\\ \; & \times \rho_{\left(d\right)}^{3}sinc^{2}\left(\rho_{\left(d\right)}\left(k-n\right)\right)sinc^{2}\left(k^{\prime }\rho_{\left(p\right)}-n\rho_{\left(d\right)}\right)\\ V_{I_{i_{3}}}= & \frac{E_{b}^{2}}{4\beta^{2}}\sum_{p=1}^{U}\sum_{p^{\prime }=1,p^{\prime }\neq p}^{U}\sum_{n=1}^{\beta }\sum_{k=-2}^{\beta +2}\sum_{k^{\prime }=-\infty }^{\infty }\sum_{l=1}^{L}\sum_{l^{\prime }=1}^{L}{\left(\alpha_{i,l}^{p}\alpha_{i^{\prime },l^{\prime }}^{p^{\prime }}\right)}^{2}\\ \; & \times \rho_{\left(p\right)}\rho_{\left(p^{\prime }\right)}\rho_{\left(d\right)}^{2}sinc^{2}\left(k\rho_{\left(p\right)}-n\rho_{\left(d\right)}\right)\times sinc^{2}\left(k^{\prime }\rho_{\left(p^{\prime }\right)}-n\rho_{\left(d\right)}\right). \end{array}$$

The noise variance is equally affected by the sinc filter at the reception. The result shown in [28] (Eq. 3.3) is valid for our case since the noise is only affected by the filter of the desired user at detection. As a result, the power of the noise is reduced by a factor of $G_{k,n}$ for the sampling parameter $\rho_{\left(d\right)}$ as:

(25) $$\begin{array}{cc} Cov(w_{k},w_{n})= & \sigma^{2}G_{k,n}\\ = & \sigma^{2}\rho_{\left(d\right)}sinc\left(\rho_{\left(d\right)}\left(k-n\right)\right), \end{array}$$

where σ is the standard deviation of the noise. Henceforth, for a noise with a double-sided power spectral density $N_{0}$, the variance of the noise terms is expressed as

(26) $$\begin{array}{cc} V_{N_{i}}= & \frac{N_{0}E_{b}}{2\beta }\sum_{n=1}^{\beta }\sum_{k=-2}^{\beta +2}(\sum_{k^{\prime }=-\infty }^{\infty }\sum_{l=1}^{L}{\left(\alpha_{i,l}^{d}\right)}^{2}\rho_{\left(d\right)}^{3}sinc\left(\rho_{\left(d\right)}\left(k-n\right)\right)sinc^{2}\left(\rho_{\left(d\right)}\left(k^{\prime }-n\right)\right)\\ \; & +\frac{N_{0}E_{b}}{2\beta }\sum_{p=1}^{U-1}\sum_{n=1}^{\beta }\sum_{k^{\prime }=-\infty }^{\infty }\sum_{l=1}^{L}{\left(\alpha_{i,l}^{p}\right)}^{2}\rho_{\left(p\right)}\rho_{\left(d\right)}^{2}sinc\left(\rho_{\left(d\right)}\left(k-n\right)\right)sinc^{2}\left(k^{{}^{\prime }}\rho_{\left(p\right)}-n\rho_{\left(d\right)}\right)\\ \; & +\frac{N_{0}^{2}}{4}\sum_{n=1}^{\beta }\rho_{\left(d\right)}^{2}sinc^{2}\left(\rho_{\left(d\right)}\left(k-n\right)\right)). \end{array}$$

For non-coherent chaos-based communications in regular Nyquist transmissions, the covariance is often neglected while expressing variances, given the low cross-correlation between chaotic sequences when time delayed or for different chaos generators,

(27) $$x_{\left(i,t\right)}.{\left(x_{\left(j,t\right)}\right)}^{T}\approx 0\;i\neq j.$$

However, in sub-Nyquist transmissions with the introduction of the sinc terms as seen in the derivation of the variances and the expectation, covariances are introduced in the formulation of the BER expression. Here, we explore the different covariances that may occur in this scenario of multi-user chaos based transmission below the Nyquist rate.

For the Chebychev map [29], we know that the joint PDF of two chaotic variables $x_{0}$ and $x_{1}$, time delayed by *t*, is expressed as

(28) $$f\left(x_{0},x_{1};t\right)=\pi^{-1}{\left(1-x_{0}\right)}^{-\frac{1}{2}}\delta \left(x_{1}-T_{k^{t}}\left(x_{0}\right)\right),$$

where $T_{k^{t}}$ is the Chebychev polynomial for a variable *x* expressed as

(29) $$T_{k^{t}}\left(x\right)=\cos\left(k.\arccos\left(x\right)\right),$$

where cos is the cosine function and δ is the delta function.

The variance of a sum of dependent random variables $X_{i}$ with weights $a_{i}$ is known as [30],

(30) $$\begin{array}{cc} var(\sum_{i=1}^{n}a_{i}X_{i})= & \sum_{i=1}^{n}a_{i}^{2}var\left(X_{i}\right)+2\sum_{i=1}^{n}\sum_{j=i+1}^{n}a_{i}a_{j}cov\left(X_{i},X_{j}\right), \end{array}$$

and the covariance is given as

(31) $$cov\left(X_{i},X_{j}\right)=E\left(X_{i}.X_{j}\right)-E\left(X_{i}\right)E\left(X_{j}\right).$$

Given that the chaotic sequences are generated with zero mean (*E*($X_{i}$) = 0), the covariance of two FTN filtered chaotic sequences is derived as

(32) $$\begin{array}{cc} Cov(X_{i},X_{j}) & =E(X_{i}.X_{j})\\ \; & =E(\int_{-\infty }^{\infty }\int_{-\infty }^{\infty }x_{i}.x_{j}.G_{T}^{i}\left(t\right)G_{T}^{j}\left(s\right)dtds)\\ \; & =\int_{-\infty }^{\infty }\int_{-\infty }^{\infty }E\left(x_{i}.x_{j}\right).G_{T}^{i}\left(t\right)G_{T}^{j}\left(s\right)dtds, \end{array}$$

where $G_{T}^{i}\left(t\right)G_{T}^{j}\left(s\right)$ are two sub-Nyquist filters from two random users with different sampling rates. We use the joint PDF of two chaotic variables to compute the expectation $E(x_{i}.x_{j})$

(33) $$\begin{array}{cc} E(x_{i}.x_{j}) & =\int_{-\infty }^{\infty }\int_{-\infty }^{\infty }x_{i}.x_{j}.f_{x_{i}x_{j}}\left(x_{i},x_{j}\right)dx_{i}.dx_{j}\\ \; & =\int_{-\infty }^{\infty }\int_{-\infty }^{\infty }x_{i}.x_{j}.\pi^{-1}{\left(1-x_{i}^{2}\right)}^{-\frac{1}{2}}\delta \left(x_{j}-x_{i}\right)dx_{i}.dx_{j}\\ \; & ={\left(\frac{E_{b}}{\beta }\right)}^{2}\int_{-1}^{1}\pi^{-1}{\left(1-x_{i}^{2}\right)}^{-\frac{1}{2}}\\ \; & =\frac{E_{b}^{2}}{2\beta^{2}} \end{array}$$

As a result, by injecting the expectation into (32) and using the results from (7), the new covariance introduced by the FTN regime is expressed in terms of the detection matrix $G_{k,n}$ as follows

(34) $$\begin{array}{c} Cov\left(X_{i},X_{j}\right)=\frac{E_{b}^{2}}{2\beta^{2}}\sum_{n=1}^{\beta }\sum_{k=-2}^{\beta +2}G_{k,n}. \end{array}$$

This covariance occurs even in the absence of noise and for single-user cases, and is strictly inherent to the nature of the filters used for transmission/reception.

Finally, the overall BER expression of the MU-FTN-DCSK system over an AWGN channel is expressed identically to DCSK as

(35) $$\begin{array}{c} \mathrm{BER}\left(\mathrm{SNR}\right)=\frac{1}{2}erfc\left(\frac{2\sum Var}{E^{2}}\right) \end{array}$$

Hence, using the derived expressions of expectation and variances, the BER expression over the AWGN channel can be obtained by assuming unitary channel coefficients i.e., $\alpha_{i,l}=1,\;\forall \;i,l$. Thus, the BER performance over multi-path Rayleigh fading channels is expressed as

(36) $$\begin{array}{c} \mathrm{BER}\left(\gamma \right)=\int_{\gamma =-\infty }^{\infty }\frac{1}{2}erfc\left(\frac{2\sum Var}{E^{2}}\right)f\left(\gamma \right)d\gamma . \end{array}$$

## 4. Numerical Analysis and Results

This section presents the MU-FTN-DCSK system’s simulation results over AWGN and multi-path Rayleigh fading channels. For the Rayleigh channel, we consider an L=3 multi-path channel model with power levels of $\left\{P_{1}=\frac{7}{10},P_{2}=\frac{2}{10},P_{3}=\frac{1}{10}\right\}$, with $P_{i},i=1,\ldots 3$ being the variance of the channel gains $\alpha_{i,l}$. Due to the high computation time dedicated for the sinc filter at transmission, since a continuous-time signal is simulated with many points for processing, the transmitter is implemented in C++ to use the multi-thread and multi-processors features. For this purpose, the Intel MKL library is used to implement the filter and other vector manipulations. Moreover, a comparison with state-of-the-art modulations is carried out to establish the proposed system’s relative performances.

### 4.1. BER Analysis

**Figure 2:**
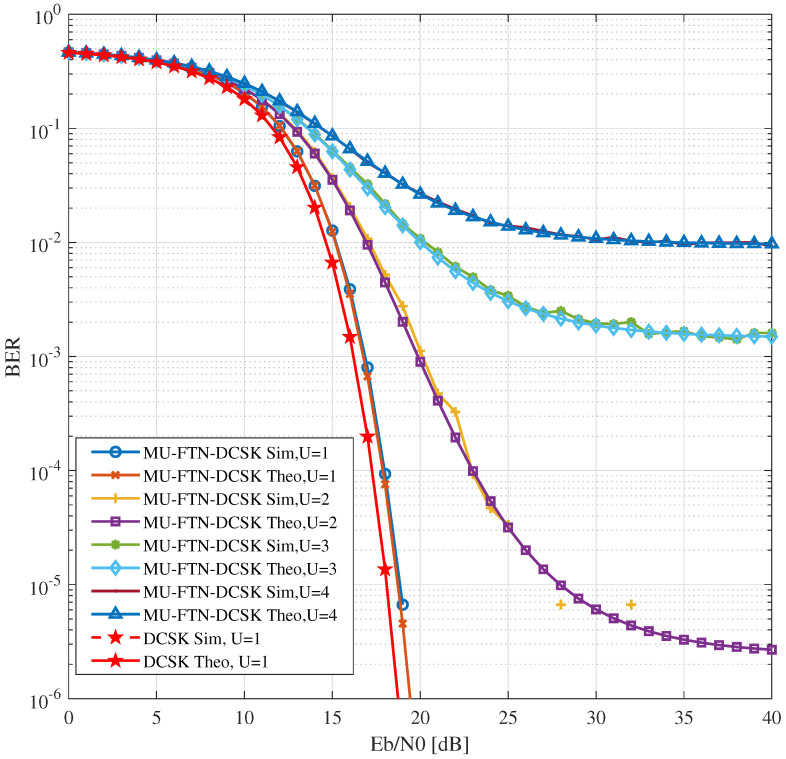
BER performance of MU-FTN-DCSK over AWGN channel with different number of users *U* = {1,2,3,4}, β=150, $\rho_{\left(d\right)}=0.9$, $\rho_{\left(p\right)}=\left\{0.8,0.7,0.6\right\}$.

Figure 2 presents BER results for different numbers of users over the AWGN channel. The parameters used for the simulations are β=150, $\rho_{\left(p\right)}=\left\{0.9,0.8,0.7,0.6\right\}$ where the main user always takes the value of $\rho_{\left(d\right)}=0.9$. We can notice the tiny gap for a single user transmitting at a sub-Nyquist rate ($\rho_{\left(d\right)}=0.9$) in comparison with normal Nyquist transmissions ($\rho_{\left(d\right)}=1$), labeled as the normal DCSK, due to the ISI introduced by the sinc filter functioning in sub-Nyquist regime. For U=1 the ISI introduced by the matched filters results in an approximate 1dB loss of BER, but with a gain of 10% of the bandwidth. This means that slightly raising the energy for transmission, in order to compensate for the 1 dB, would allow a gain of 10% in bandwidth usage in both the reference and the data sequences. The analytical results match the simulation results proving the validity of the new covariance derived in (34).

The same observation is perceived when the number of users is increased. We notice that up to three users with different sampling parameters can be accepted in the system with an acceptable BER for noisy environments. In the setting used for the simulations that lead to these results with sampling parameters $\rho_{\left(p\right)}=\left\{0.9,0.8,0.7\right\}$, we obtain a slot of length (0.6 × β) of extra bandwidth. As a comparison, for *U* = 4 users, the BER performance offered by the MU-FTN-DCSK over an AWGN channel matches that of the regular DCSK over the Nakagami-m fading for a channel parameter m=0.5 [3] due to the severe interference caused by the simultaneous transmissions of all users in the sub-Nyquist regime. Nonetheless, the bandwidth gain here is 2β if we sum the individual gains from different users transmitting at a sub-Nyquist rate in both the reference and data signals. An optimization in the choice of the spreading factor would improve these results since it can significantly contribute to the reduction of ISI.

**Figure 3:**
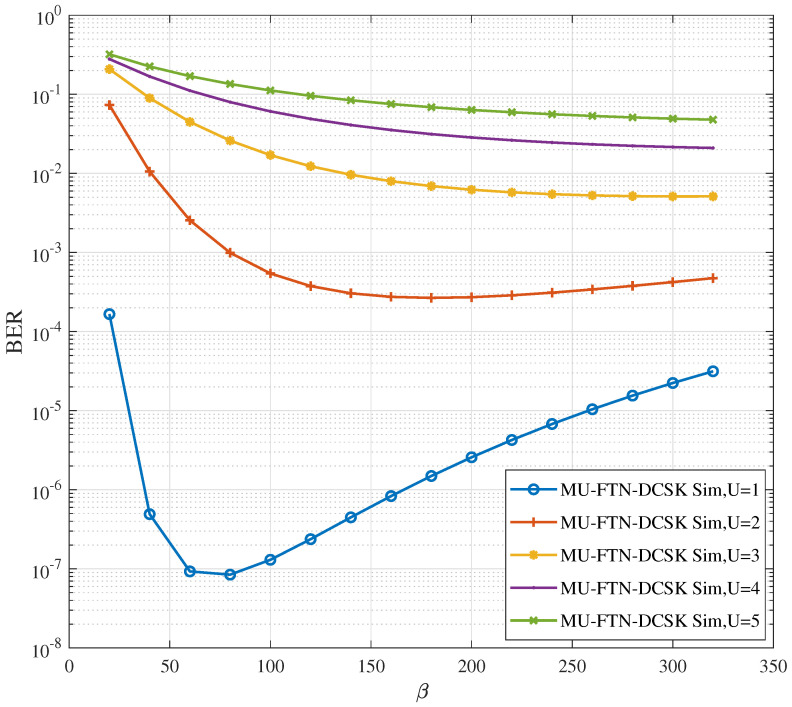
BER performance of MU-FTN-DCSK over AWGN channel for different spreading factors and number of users *U* = {1,2,3,4}, β=150, $\rho_{\left(d\right)}=0.9$, $\rho_{\left(p\right)}=\left\{0.8,0.7,0.6,0.5\right\}$, and SNR = 25 dB.

In this sense, Figure 3 provides insights into the impact of the spreading factor relative to the BER performances of the MU-FTN-DCSK system for an SNR = 25 dB. The sampling parameter takes the values $\rho_{\left(p\right)}=\left\{0.9,0.8,0.7,0.6,0.5\right\}$ with $\rho_{\left(d\right)}=0.9$ for the desired user. As observed in this figure, increasing β positively impacts the system’s capability to reduce the ISI for a certain range before reaching a plateau of BER performances centered around certain values depending on the number of users under consideration. Notably, after these specific values, higher BER values are observed. However, the value of β is also limited by the desired data rate, which creates a trade-off. We also notice that a system with a superior number of users and higher spreading factors achieves the same BER as a system with fewer users with reduced spreading factors for certain values of BER. Hence, trading the two configurable parameters for optimal access is possible with the new design of MU-FTN-DCSK.

**Figure 4:**
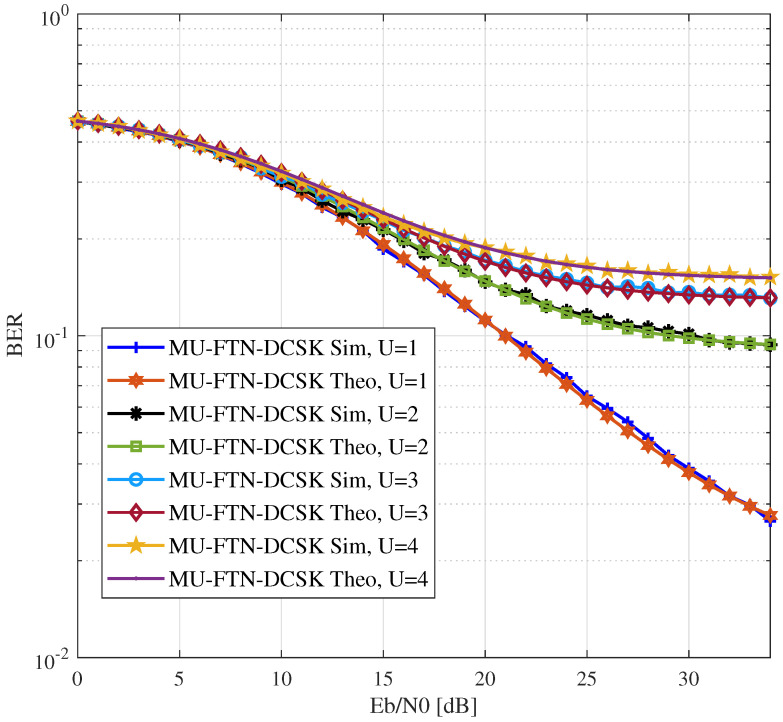
BER performance of MU-FTN-DCSK over Rayleigh channel for a dissimilar power distribution, *U* = {1,2,3,4}, β=150, $\rho_{\left(d\right)}=0.9$, $\rho_{\left(p\right)}=\left\{0.8,0.7,0.6\right\}$.

The BER performance of the MU-FTN-DCSK over multi-path Rayleigh fading channels with dissimilar channel gains is presented in Figure 4. We can notice from the BER performance of a single user that the degradation caused by the ISI is comparable in magnitude to the degradation for the AWGN situation. However, now the impact of the floor introduced by the extra Chaos-Chaos interference on the BER, derived in (34), is more apparent, regardless of the number of users in the system. Nonetheless, it can be seen that such high ISI has a nearly identical impact on the BER for the numbers of users superior to two. Moreover, the BER performances in this figure confirm the system’s capability to combat multi-path loss.

**Figure 5:**
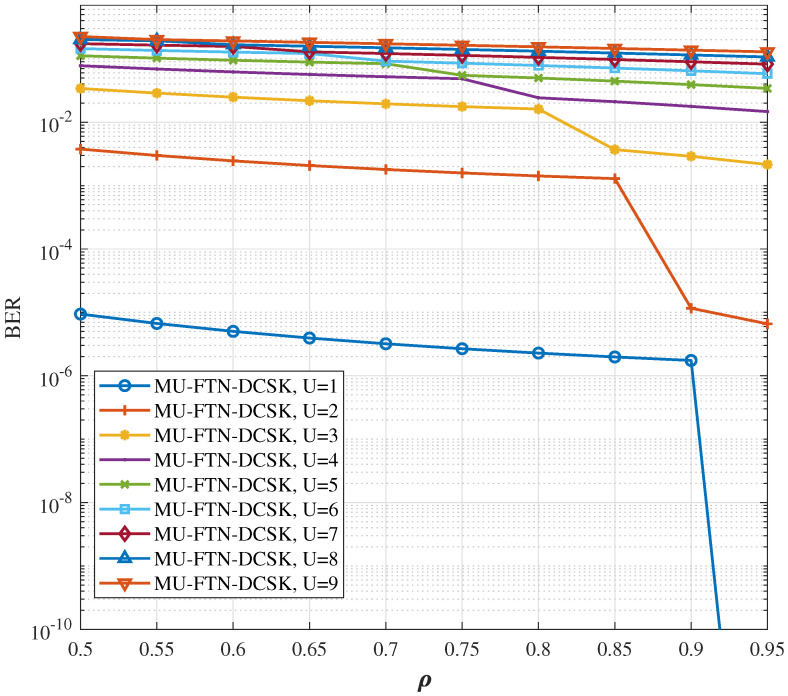
BER performance for different sampling rates and number of users over the AWGN channel, *U* = {1,…9}, β=100, $\rho_{\left(p\right)}=\left\{0.95,0.9,\ldots 0.5\right\}$, SNR = 30 dB.

**Figure 6:**
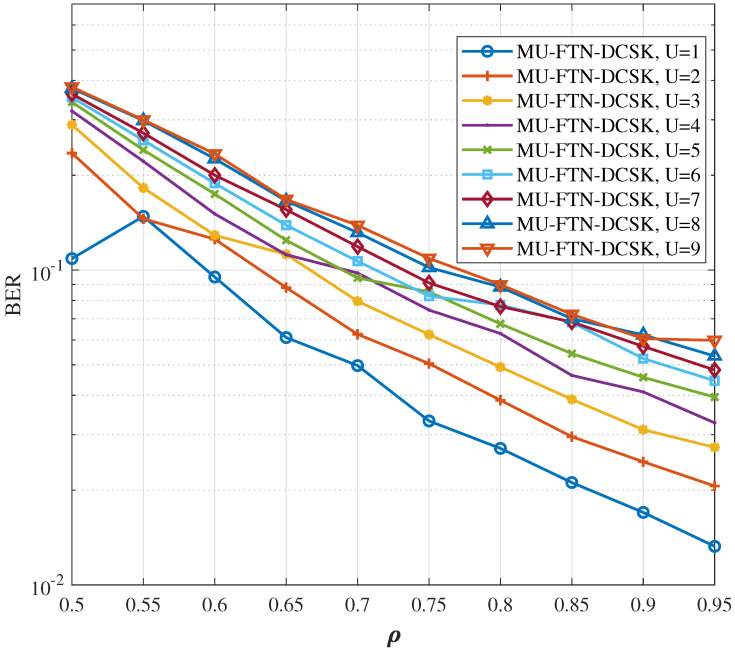
BER performance for different sampling rates and number of users over the dissimilar multi-path Rayleigh fading channel, *U* = {1,…9}, β=100, $\rho_{\left(p\right)}=\left\{0.95,0.9,\ldots 0.5\right\}$, SNR = 30 dB.

The impact of the sampling rate on the BER performances over AWGN and multi-path Rayleigh fading channels is presented for different numbers of users up to a maximum *U* = 9 in Figure 5 and Figure 6, respectively. An SNR of 30 and a sampling parameter ρ of [0.05, …, 0.95], are used for the primary user, while the other user’s sampling parameters are switched in the same range of values, but different from the desired user’s sampling parameters in order to enable detection. It is interesting to notice that for a single user, in the high SNR regime, the reduction in sampling rate to half the Nyquist limit (ρ=0.5) does not hinder the MU-FTN-DCSK from having a successful transmission with a good BER performance, especially for the AWGN channel. The same observation can be applied for up to three users in the system with relatively close sampling rates but for a reduced spectral efficiency. As a result, the ISI, despite impacting the system’s performance considerably even in the absence of noise, does not prevent a successful transmission, even with a substantial decrease in sampling rates. Therefore, the combination of DCSK with FTN seems very promising in improving the spectral efficiency if the detection can be improved.

**Figure 7:**
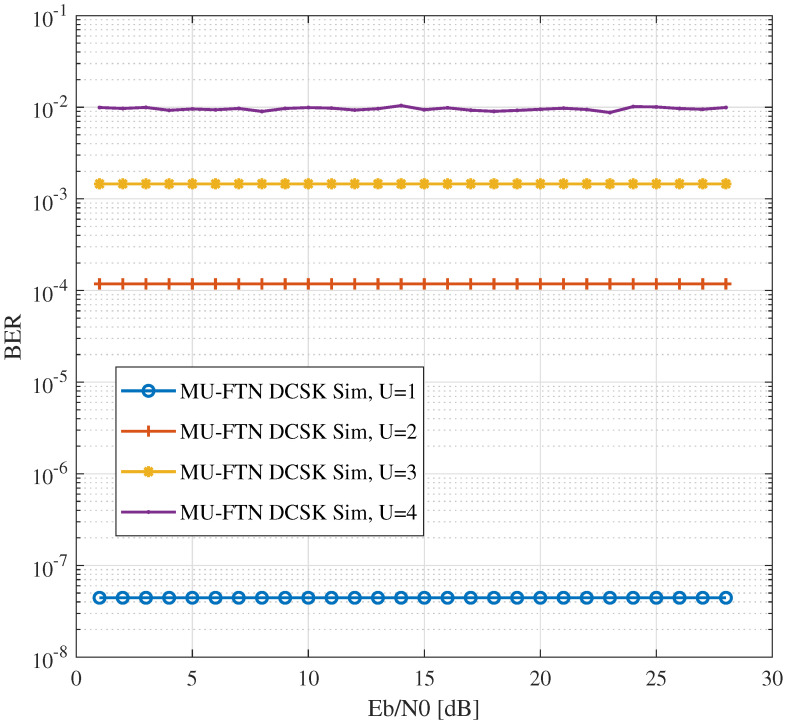
BER performances of MU-FTN-DCSK for a noiseless channel.

The BER performance of the MU-FTN-DCSK system is dictated by the extra interference between the chaotic signals, which does not exist in traditional chaos-based systems. The chaos-chaos interference is caused solely by the functioning of the sub-Nyquist filters in a similar manner to the ISI. The impact of this new form of interference can be seen in Figure 7, where a noise-free channel ($N_{0}=0$) is considered for the transmission. As seen here, the chaos-chaos interference results in a performance floor that establishes the best BER performance that can be obtained for a given number of users. The new form of interference depends on the filter and its sampling rate and is also cumulative to the group of users in the system.

**Figure 8:**
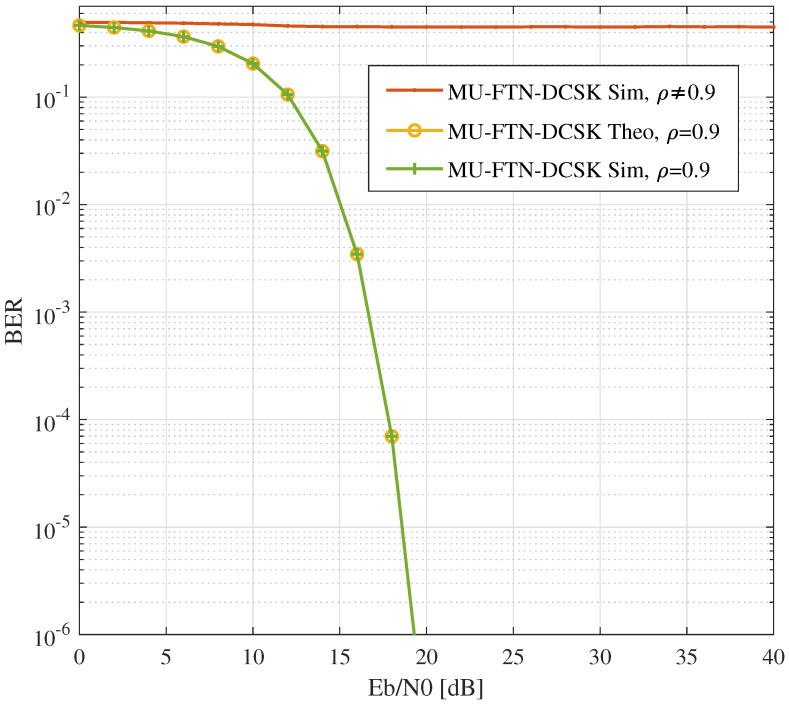
BER performances of MU-FTN-DCSK for a faulty sampling rate.

The introduction of the custom-selected sampling rates to enable FTN and multi-user transmission results in an additional feature presented by the MU-FTN-DCSK in terms of security. Indeed, the probability of detection goes to zero if the appropriate sampling rate is not used as presented in Figure 8. Using a sampling rate different from the desired users’ rate enables perfect secrecy, as seen in the simulation results in Figure 8 for $\rho_{\left(d\right)}\neq 0.9$. This intuitively comes down to the fact that the use of an erroneous sampling rate subsequently alters the capability of detection of the basic DCSK design, given that the value of β changes by the change of the sampling rate. Hence, MU-FTN-DCSK can be seen as an improved version of DCSK in terms of security since it requires an extra parameter for successful detection.

### 4.2. Performance Comparison

The MU-FTN-DCSK system is compared to newly introduced DCSK-based schemes in order to evaluate its effectiveness as a newly proposed design. In terms of BER performances, the system lags due to the impact of the interferences caused by the functioning of the filter in the FTN regime. For instance, for a multi-path Rayleigh fading channel, a difference of 5 dB is observed in comparison with the CIM-MC-M-DCSK MISO-SWIPT system [31]. However, the complexity is greatly reduced since the latter system uses many additional signal processing transforms to accomplish the given gain. Table 1 gives a detailed comparison of the complexity of the two systems. Moreover, the BER perfromance of MU-FTN-DCSK is comparable to the performance of the GCI-DCSK SWIPT for the AWGN and Rayleigh fading channels. The MU-FTN-DCSK also presents an overall better BER relative to the spreading factor for the single user scenario.

In the addition to the lesser complexity, the MU-FTN-DCSK offers a better spectral efficiency given the same configuration as the CIM-MC-M-DCSK MISO-SWIPT system. For example, for the CIM-MC-M-DCSK MISO-SWIPT system and for a number of antennas *N* = 1 and a modulation order *M* = 2, the spectral efficiency is lower than that of the MU-FTN-DCSK system for any value of the sampling parameter meeting the condition $\rho_{\left(p\right)}<0.65$. In addition, the accumulative spectral efficiency gained from all users transmitting in the FTN regime can be higher than systems having very advanced designs. Furthermore, the results above indicate that using FTN in combination with additional signal processing methods can result in much higher spectral efficiency gains.

## 5. Conclusions and Recommendation

A multi-user Faster than Nyquist DCSK system, where we use transmission/reception filters below the Nyquist required limit to enable a gain in spectral efficiency as well as simultaneous access for multiple users, was presented in this paper. FTN, in combination with DCSK, enables the enhancement of the latter in terms of bandwidth usage, which is one of the main drawbacks of traditional DCSK designs. The newly proposed system enables multi-user access without the addition of complicated blocks or new procedures. Using different sampling rates in FTN is a simple yet very effective design showing promising results. Moreover, several new algorithms have been proposed for optimal FTN detection, which can be added to this conceptual system for an improved performance and may even be able to rival coherent chaos-based systems like Chaos Shift Keying (CSK). The performance analysis of the newly proposed system has shown a possible multiple-access of up to three users with good BER results even without additional transmission power or a high spreading factor. Besides, it presents the same resistance to multi-path fading as the traditional DCSK systems while going below the Nyquist rate and for multiple users. The newly designed system allows a gain in bandwidth between 10% and 200% of the DCSK reference size, assuming up to four users (U=4), and can go beyond if a better detection algorithm is introduced. One user’s gain can vary depending on the sampling parameter value, from 24% at the Mazo limit up to 40% when ρ=0.6. The main challenge encountered during this study was the use of lower sampling rates, as this increases the level of ISI. Moreover, adding new users results in higher levels of IUI which restricts the number of users in the system. The limitation in the number of users or the minimum sampling rates to consider was shown here to be related to the new form of interference between chaos signals that happens to originate from the usage of the filters on non-orthogonal transmissions. Hence, future works will be directed towards improving the detection algorithm in order to eliminate interference and the possibility of applying both time and frequency FTN transmissions. In addition, MIMI-OFDM might be a possible approach in improving BER performances of the current design while keeping the design simple.

## Figures and Tables

**Table 1:** Complexity comparison.

Modulation	MU-FTN-DCSK	CIM MC-M-DCSK
		MISO-SWIPT
Blocks	matched filters	matched filters
		serial to parallel
		bit/symbol converter
		code index modulation
		Hilbert filter
		code index modulation
		Walsh codes

## Data Availability

Not applicable.
